# The combination of CTCs and CEA can help guide the management of patients with SPNs suspected of being lung cancer

**DOI:** 10.1186/s12885-020-6524-1

**Published:** 2020-02-10

**Authors:** Jian Zheng, Xiong Ye, Yanan Liu, Yuxia Zhao, Mudan He, Hui Xiao

**Affiliations:** 1 Department of Thoracic Surgery, Shanghai General Hospital, Shanghai Jiaotong University, Shanghai, China; 2 College of Clinical Medicine, Shanghai University of Medicine & Health Science, Shanghai, China; 3 Department of Clinical Laboratory, Shanghai Children’s Hospital, Shanghai Jiaotong University, Shanghai, China; 4 Department of Respiratory and Critical Care Medicine, Shanghai First Hospital of Baoshan Branch, Shanghai, China; 5 Department of Respiratory and Critical Care Medicine, Shanghai General Hospital, Shanghai Jiaotong University, 85Wujin Road, Shanghai, 200080 China

**Keywords:** Circulating tumour cells, CEA, Solitary pulmonary nodules, Lung cancer, Management

## Abstract

**Objective:**

Solitary pulmonary nodules (SPNs) is a common radiographic finding and require further evaluation because of the possibility of lung cancer. This study aimed to determine the sensitivity and specificity of circulating tumour cells (CTCs) as a marker for the diagnosis of SPNs and the integration of CTCs, carcinoembryonic antigen (CEA) and imaging findings to improve the sensitivity and specificity of diagnosis in patients with SPNs suspected of being lung cancer.

**Method:**

For the serum biomarker assay, the concentration of CEA was measured by an automated electrochemiluminescence analyzer. CTCs were collected from 6 ml of blood by the SE i-FISH method, which detects the gene copy number in eight chromosomes and the tumour-associated antigen CK18.

**Results:**

With a threshold of 6 CTC units, the method showed a sensitivity of 67.1% and a specificity of 56.5% in the diagnosis of NSCLC, especially in the upper lobe, in which the diagnostic strength was the highest (*P* < 0.01). CTCs, CEA and nodule type had the highest diagnostic efficacy (area under the curve, 0.827; 95% confidence interval, 0.752–0.901) in patients with SPNs being suspected lung cancer. Combining CTCs (cut-off value 12 units) with CEA (1.78 ng/ml), the method showed a sensitivity of 77.8% and a specificity of 90% in the diagnosis of NSCLC, especially in the upper lobe, subsolid nodules and nodules ≥8 mm.

**Conclusions:**

Our results demonstrated that CTCs are feasible diagnostic biomarkers in patients with SPNs, especially in the upper lobe. Furthermore, CTCs combined with CEA showed higher diagnostic efficacy in the upper lobe, subsolid nodules and nodules ≥8 mm.

## Highlights

CTCs as a marker for the diagnosis of SPNs. The integration of CTCs, CEA and imaging findings improve the sensitivity and specificity of diagnosis in patients with SPNs suspected of being lung cancer.

## Background

Circulating tumour cells (CTCs) analysis has recently emerged as a liquid biopsy approach for the early diagnosis [1, 2], biomarker [3], evaluation of curative efficacy [4], evaluation of relapse [5] and prognostic prediction [6, 7] in several solid tumours, including breast cancer [8, 9],gastric cancer [10], prostate cancer [11], head and neck cancer [12], bladder cancer [13], and lung cancer [14–16]. CTCs also showed the potential efficacy in metastasis cancer, such as metastatic breast cancer [17, 18] and colorectal cancer [19].

Carcinoembryonic antigen (CEA) was first identified in 1977 as a marker of tumour extent and response to treatment in patients with lung cancer [20]. Subsequently, CEA became one of the most widely used tumor markers [21, 22]. Two-marker combinations are more suitable than multi-marker combinations for the serological screening of tumours, especially the combination of CEA and CA125 in healthy subjects [23], although the sensitivity and specificity of the combination of CTCs and CEA for the diagnosis of solitary pulmonary nodules (SPNs) suspected of being lung cancer are unknown.

SPNs are relatively common; SPNs are defined as a single, well-circumscribed, radiologic opacities that measure up to 3 cm in diameter and are surrounded completely by aerated lung [24]. SPNs need further evaluation because of the possibility of lung cancer. Most patients suffering from indeterminate pulmonary nodules undergo sequential computed tomography (CT) studies. Low-dose computed tomography is an effective means of early diagnosis and screening, which could reduce mortality due to lung cancer by 20% [25]. Despite follow-up and treatment of nodules according to the guidelines, the high rate of false positives of small nodules still represents a distinct clinical challenge. A more efficient and effective strategy that would avoid delays in diagnosis, decrease radiation exposure and reduce the need for invasive procedures is urgently needed to manage patients with pulmonary nodules.

In our study, the relationships among CTCs, CEA, and SPNs are reported in detail. This study aimed to determine the sensitivity and specificity of CTCs and the combination of CTCs and CEA in the diagnosis of SPNs, especially those suspected of being lung cancer.

## Patients and methods

### Patients selection

We recruited 161 patients with SPNs who undergoing treatment were from January 2017 to September 2018 for enrolment in the study. The diagnosis of SPNs was based on assessments of low-radiation computed tomography scans by two radiologists. All patients received established diagnoses by pathologic examination. The inclusion criteria were the presence of one solid SPN, which is defined as a nodule with at least a solid component > 80% of the total volume, and available CT scan encompassing the lungs and a definitive diagnosis by means of tissue biopsy or imaging follow-up, as suggested by guidelines [26]. The exclusion criteria were the presence of visible nodule calcifications and the presence of more than one nodule in the same patient. No patient underwent preoperative radiotherapy, chemotherapy or any other treatment. Disease stages were based on the eighth edition of the American Joint Committee on Cancer staging manual. Surgery was performed under the supervision of the three thoracic surgeons at the thoracic center, and all operations were performed as two-hole thoracoscopic lung nodule wedge resections or lobectomy. The demographics and diagnoses of the patients are shown in Table 1.

**Table 1:** Detailed patient demographics and diagnoses

Characteristics	No.
Lung cancer (*N* = 85)	
Age, years; mean (range)	62.1 (28–80)
Gender (male/female)	36/49
MTD,mm; mean (range)	17 (5–30)
Histology (number of patients)	
ADC	71 (83.5%)
ACIS	12 (16.9%)
MIA	25 (35.2%)
ADI	34 (47.9%)
Adenosquamous cell carcinoma	1 (1.2%)
SCC	6 (7.1%)
LCC	3 (3.5%)
Small cell carcinoma	1 (1.2%)
Unknown	3 (3.5%)
Benign diseases (*N* = 46)	
Age, years;mean (range)	56.5 (23–85)
Sex (male/female)	21/25
MTD mm; mean (range)	11.1 (3–30)
Inflammatory nodules	19 (41.3%)
Hamartoma	4 (8.7%)
Pulmonary tuberculosis	1 (2.2%)
Inflammatory pseudotumour	2 (4.3%)
Lymph nodes	1 (2.2%)
Lipoma	1 (2.2%)
Granuloma	1 (2.2%)
Organized pneumonia	1 (2.2%)
Abscess	1 (2.2%)
Leiomyomata	2 (4.3%)
Fungal infection	1 (2.2%)
Others	12 (26.1%)

*ADC* adenocarcinoma; *MTD* maximum tumour diameter; *ACIS* adenocarcinoma in situ; *MIA* microinvasive adenocarcinoma; *ADI* adenocarcinoma infiltrating; *SCC* squamous cell carcinoma; *LCC* large cell carcinoma

### Study design

This retrospective trial was performed at a clinical center in the Department of Respiratory and Critical Care Medicine and Thoracic Surgery in our hospital to assess the number of CTCs, the concentration of CEA and the nodule characteristics on chest CT to predict the likelihood that SPNs were cancerous. The primary inclusion criteria were measurable nodules on the imaging examinations and the availability of data regarding CEA and CTCs. The Institutional Review Board at Shanghai General Hospital, Shanghai Jiaotong University, approved the current retrospective study and informed written consent was obtained from all subjects before the study commenced.

### Detection of CTCs

The detection of CTCs was performed as described previously by Liu et al. and Ge et al. [27, 28] In briefly, CTC enrichment and identification was performed according to the instructions of the Cytelligen CTC Enrichment Kit and Human Tumor Cell Identification Kit (Cytelligen, San Diego, CA, USA). Blood samples were collected in 6-ml ACD-containing tubes from an antecubital vein in all patients before surgery. The technicians who performed the CTC detection tests were blinded to the sample identification. All samples had been de-identified before the technicians received them. After the blood samples were centrifuged, the white buffy was collected and mixed with a tumor-associated antigen CK18 (showing that the captured cells were epithelial cells) and the CD45 antibody (demonstrating that the captured cells derived from non-leukocytes). After shaken, the mixture was subjected to magnetic separation. The resulting cell pellet was mixed with 100 μl of fixative and used to coat CTC slides. A Centromere Probe (CEP 8) Spectrum Orange (Vysis, Abbott Laboratories, Abbott Park, IL, USA) was added to the slides, denatured at 76 °C for 10 min and hybridized at 37 °C for 3 h. The slides were incubated for 2 h in the dark at room temperature. After being washed twice, 5 μl of mounting medium(Ultra Cruz, Santa Cruz Biotechnology, CA, USA) staining with 4′, 6-diamidino-2-phenylindole (DAPI, showing the karyotypes of the captured cells) was added, and then, the cell number was counted under a Carl Zeiss Axioplan 2 imaging fluorescence microscope (ZEISS Company, Germany). The identification of CTC was DAPI+/CK18+/CD45−/CEP8 ≥ 3pots. The count was repeated 3 times, and the mean was selected as the final value for each patient.

### Measurement of CEA

Peripheral venous blood samples (3.0 ml) were collected from patients prior to surgery. The serum was separated by centrifugation at 4000 rpm for 10 min within 2 h. The concentration of CEA was measured by an automated electrochemiluminescence analyzer (Roche Diagnostics, Bavaria, Germany). All tests were performed according to the protocols in the instrument operating manual. CEA values obtained within ±7 days of the date of a given CTC measurement were included.

### Imaging

Computed tomography imaging scans of the chest were first performed to identify lung nodules. The images were assessed by two certified radiologists, including nodule margin, nodule diameter, nodule density, burr sign, pleural traction, and vascular bundle sign. Finally, the thoracic surgeon decided whether to perform an operation.

### Statistical analysis

The CTC units are presented as medians and interquartile ranges. Mann-Whitney U tests were used to evaluate the significance of differences between two groups, and Kruskal Wallis tests were used to compare the numbers of CTCs among three or more groups. The best cutoff values to discriminate patients with lung cancer from those with benign lung lesions were identified using receiver operating characteristic (ROC) curve analysis, and the area under the ROC curve (AUC) was calculated for each index. The Youden index was used to identify the optimal cutoff point and diagnostic efficiency. All tests of statistical significance were two-sided, and *p* < 0.05 was considered to indicate a statistically significant difference. The statistical analyses were performed with SPSS 16.0 software (SPSS Inc., Chicago, IL), Prism 5.0 (GraphPad Software Inc., San Diego, CA) and MedCalc 15.2 software.

## Results

### Patient characteristics

Between January 2017 and July 2018, 161 patients were enrolled, 131 of whom met the inclusion and exclusion criteria and were assessable for the objectives pertaining to lung cancer or benign nodules. At the time of these analyses, the two groups had similar demographic characteristics (age, gender, and smoking). Patient characteristics are listed in Table 1.

### CTC levels in clinical samples with SPN and radiographic imaging results

**Fig. 1:**
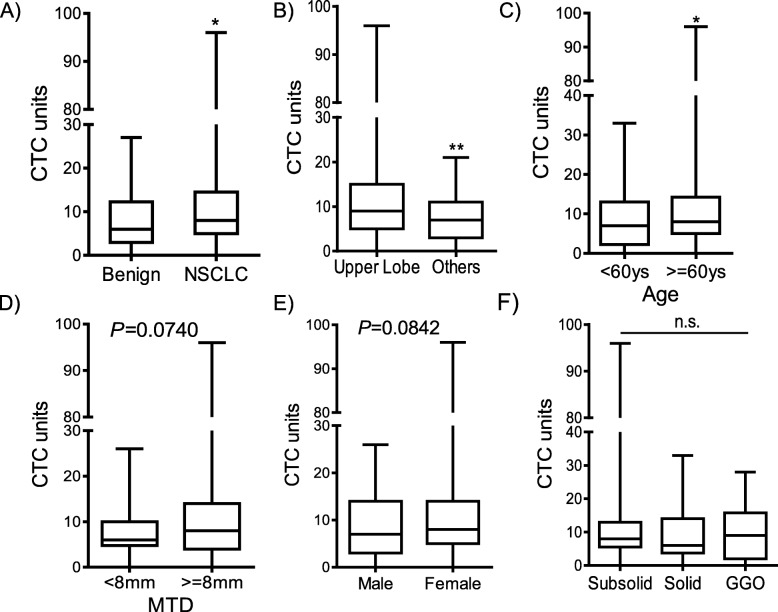
CTC counts in patients with SPNs. (**A**). CTC counts between patients with benign lung diseases and those with NSCLC. (**B**). CTC counts in patients with nodules in the upper lobe of the lung and other sites. (**C**). CTC counts in patients with NSCLC aged < 60 years and ≥ 60 years. (**D**) CTC counts in patients with NSCLC with MTD < 8 mm and those with MTD ≥8 mm. (**E**). CTC counts in male and female patients with NSCLC. (**F**). CTC counts in patients with NSCLC with subsolid, solid and GGO nodules. (*p < 0.05.**p < 0.01)

The aim of the study was to facilitate decision-making for patients with SPNs suspected of being lung cancer. As presented in Fig. 1a, the median numbers of CTC units were 5.5 (4.13–7.28) and 12.09 (7.11–15.88) in benign nodules and patients with NSCLC, respectively. Compared with patients with benign nodules, patients with NSCLC had significantly higher levels of CTC (*p* = 0.016). The CTC level was related to age (*p* = 0.041) and but not gender (*p* = 0.842). Of the 131 evaluable patients, all (100%) underwent imaging studies. The CTC level was related to nodule position (*p* = 0.0064), especially in the upper lobe than other lobes, but not nodule size (*p* = 0.074), regardless of the nodule type (Fig. 1). Table 2 summarizes the comparison of clinical characteristics and imaging results among patient with different CTC unit levels, including age at diagnosis (≤60 years vs. > 60 years), smoking, gender (male vs. female), maximum tumour diameter (MTD, < 8 mm vs. ≥8 mm), nodule site, and type of nodules (ground-glass opacity (GGO) vs. subsolid vs. solid).

**Table 2:** CTC levels in patients with SPNs

Characteristics	CTC units, median (Interquartile Range)	*P* value
Age		0.041
< 60 years	8.1 (10.5)	
≥ 60 years	12.1(9.25)	
Gender (male/female)		0.0842
Male	8.6 (11)	
Female	11.9 (9)	
Smoking		0.4802
Yes	11.8 (4.5)	
No	9.8 (6.25)	
MTD		0.0740
< 8 mm	7.8 (5.25)	
≥ 8 mm	11.1 (10)	
Nodule type		0.5064
SSN	8.7 (6.5)	
Solid	8.2 (10.25)	
GGO	10.5 (12.75)	
Site		0.0064
Upper lobe	12.5 (10)	
Other	8.3 (8)	

*SSN* subsolid nodules; *MTD* maximum tumour diameter; *GGO* ground glass opacity

**Fig. 2:**
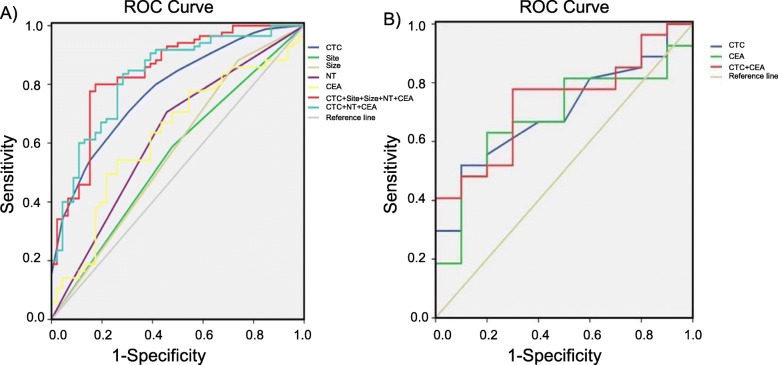
ROC curve analyses of the use of CTC, CEA and imaging examinations to differentiate patients with NSCLC from those with benign lung disease. (**A**) Combining CTC with nodule density, nodule size, nodule location, and CEA significantly improved the sensitivity and specificity of the diagnosis. Nodular density, CEA and CTC are independent prognostic factors. (**B**) The relationship between CTCs and CEA values in upper lobe, subsolid and ≥ 8-mm nodules, using the CTC threshold of 12 units/6 ml and the CEA threshold of 1.78 ng/ml

To investigate the significance of CTC levels in patients with NSCLC, the imaging and pathologic characteristics of individuals, including tumor grade (ACIS vs. MIA vs. ADI) and nodule site (upper lobe vs. others), were also tested for their associations with CTC levels. The CTC levels of patients with nodules in the upper lobe of the lung were higher than those in patients with nodules in other lobes (*p* = 0.011; Fig. 2).

### Role of CEA level, number of CTCs and imaging findings in lung cancer diagnosis and ROC analysis

**Fig. 3:**
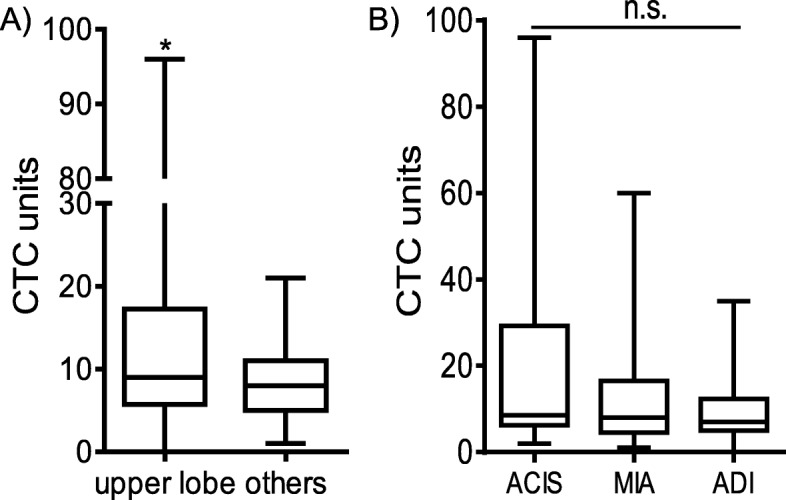
CTC counts in patients with lung cancer. (**A**) CTC counts in patients with nodules in the upper lobe of the lung and other sites. (**B**) CTC counts in patients with ADC with ACIS, MIA, and ADI. (*p < 0.05.**p < 0.01)

To further analyze the diagnostic efficiency of CTCs in patients with SPNs, we compared CTCs with CEA. With the cutoff value of 6 CTC units, the sensitivity was 67.1%, and the specificity was 56.5% in the diagnosis of NSCLC in patients with SPNs. The sensitivity of the existing clinical tumor biomarker (CEA, cutoff value 2.09 ng/ml) (54.1%) was lower than that of CTCs, but the specificity of CEA was higher (73.9%) than that of CTCs. As shown in Fig. 3a and Table 3, the CTC units had a higher AUC (0.78; 95% CI 0.70–0.86) and Youden index value (0.2358) than CEA (AUC 0.626; 95% CI 0.526–0.725). With a cutoff point for nodule size of 8 mm, the sensitivity was 88.2%, and the specificity was 73.9% in the diagnosis of NSCLC in patients with SPNs; the AUC was 0.572 (95% CI 0.466–0.677). Furthermore, with regard to the diagnosis of NSCLC in patients with SPNs, nodule type (nodule density − 600 HU) and nodule site had sensitivities of 70.6 and 58.8% and specificities of 45.7 and 47.8%, respectively.

**Table 3:** The diagnostic sensitivity and specificity of CTC, CEA, nodule type, nodule size and nodule site in patients with SPN.(*N* = 131)

Diagnostic method	Cutoff point	Sensitivity (%)	Specificity (%)	Youden Index value	AUC (95% CI)
CEA (ng/ml)	2.09	54.12	73.91	0.2803	0.626 (0.526–0.725)
CTC (units)	6	67.06	56.52	0.2358	0.780 (0.700–0.860)
Size (mm)	8	88.24	73.85	0.1432	0.572 (0.466–0.677)
NT (HU)	−600	70.57	45.73	0.2489	0.626 (0.524–0.728)
Site		58.79	47.76	0.1109	0.555 (0.451–0.659)
CTC + CEA		57.65	87.13	0.3688	0.734 (0.537–0.832)
CTC + CEA + NT		92.94	50.02	0.4290	0.827 (0.752–0.901)
CTC + CEA + NT + Size+Site		77.63	70.71	0.4829	0.841 (0.769–0.914)

*CTC* circulating tumour cell; *CEA* carcinoembryonic antigen; *NT* nodule type

The AUC of CTC combined with CEA and nodule type was 0.827, and the 95% confidence interval was 0.752–0.901. In addition, the AUC of CTC combined with CEA, nodule type, nodule size, and nodule site was 0.841, and the 95% confidence interval was 0.769–0.914, which indicates a satisfactory discrimination of patients with early-stage NSCLC from those with benign lung nodules (Fig. 3a and Table 3). Nodule type, CEA and CTC are independent prognostic factors for lung cancer.

### Roles of CEA and CTCs in the diagnosis of nodules in the upper lobe of the lung, nodules ≥8 mm, and subsolid nodules according to the ROC analysis

We also analyzed outcomes based on the number of CTCs (≥12 CTCs/6 ml) and CEA values (> 1.78 ng/ml) in the diagnosis of nodules in the upper lobe of the lung, nodules ≥8 mm, and subsolid nodules. As expected, with the cutoff values of 12 CTC units and 1.78 ng/ml CEA, the sensitivities were 51.85 and 62.96%, and the specificities were 90 and 80%, respectively. The AUCs were 0.70 (95% CI, 0.527–0.839) and 0.68 (95% CI, 0.508–0.825), respectively. The AUC for CTC combined with CEA was 0.73, and the 95% confidence interval was 0.566–0.900 (Fig. 3b and Table 4). It is noteworthy that among the patients with upper lobe, subsolid and ≥ 8-mm nodules, the combination of the number of CTCs with the CEA value resulted in a distinct improvement of the sensitivity and specificity in the diagnosis of lung cancer.

**Table 4:** The diagnostic sensitivity and specificity of CTC and CEA in patients with upper lobe, subsolid and ≥ 8-mm nodules. (*N* = 37)

Diagnostic method	Cutoff point	Sensitivity (%)	Specificity (%)	Youden Index value	AUC (95% CI)
CTC (units)	12	51.85	90.00	0.419	0.700 (0.527–0.839)
CEA (ng/ml)	1.78	62.96	80.00	0.429	0.681 (0.508–0.825)
CTC + CEA	12 /1.78	77.80	90.00	0.478	0.733 (0.566–0.900)

*CTC* circulating tumour cell; *CEA* carcinoembryonic antigen

## Discussion

The present study identified patients with suspected early-stage lung cancer detected the CTC counts and CEA levels in the peripheral blood and performed imaging examinations. The total number of CTCs was found to be influenced by the site of the SPN, the stage of the disease and the age of the patients. The present study also indicated that the number of CTC units in patients with SPNs was higher in those with early-stage NSCLC than in those with benign nodules. Furthermore, the CEA level and CTC count were found to increase the diagnostic efficiency in patients with NSCLC.

In the present study, the total number of CTCs was correlated with the stage of the disease. The number of CTCs was higher in the 85 patients with NSCLC than in the 46 patients with benign lung disease (*P* = 0.0168). There were no significant differences according to nodule size (< 8 mm vs. ≥8 mm). We believe that there should be a difference according to the nodule size and that such a difference might be detected with a modification of the methods of determining the CTC counts and more patients with SPN. Premasekharan et al proposed that by using downstream genomics in patients with metastatic lung cancer, improvements can be made in CTC isolation [29]. Although such parameters may have influenced the results, further investigations are required to assess the effect of the detection method on the number of CTCs in patients with SPNs [30, 31].

One of the key findings of the present study was that the numbers of CTCs in patients with SPNs that were early-stage NSCLC were associated with the location of the nodule in the upper lobe of the lung and the age of the patients but not the type of nodule or the pathological type. Thus, lung cancer is more commonly identified in the upper lobe of the lung [32]. However, the underlying mechanism requires further functional investigations.

The use of CTC counts for the diagnosis of NSCLC in patients with SPNs still faces challenges. There are not universal reference ranges available for all types of cancer. Therefore, we compared CTCs with the existing clinical tumor biomarker for NSCLC (CEA). With the cutoff point of 6 CTC units, the specificity was 56.5% for the diagnosis of NSCLC in patients with SPNs. Furthermore, when CTC counts were combined with CEA levels and imaging results, the AUC was 0.841, and the 95% CI was 0.764–0.914; this combination can satisfactorily discriminate patients with early-stage NSCLC from those with benign lung nodules. This finding is consistent with the results of a study by Yu et al [33], who found that with a threshold of 8.64 CTC units, FR-positive CTCs were feasible diagnostic biomarkers in patients with NSCLC. However, the study by Yu et al. used different detection methods, and there were differences in the staining protocols that were used. It is noteworthy that among the patients with suspected lung cancer (upper lobe, subsolid and ≥ 8-mm nodules), the combination of CTC count with CEA level resulted in a distinct improvement in the diagnosis of NSCLC in terms of the sensitivity and specificity; the AUC was 0.73, and the 95% CI was 0.566–0.90. The value of CEA was lower in this study than in previous studies in other institutions, which is difficult to explain. Patient diagnostic criteria and/or disease stages may have been different in previous studies.

The limitations of the present study were the small cohort size, the single-center retrospective nature of the study, and the noncomparative nature of the clinical analysis. In addition, our analysis was based on identifying the total number of CTCs rather than on the effects of CTCs in the diagnosis. Another limitation of the present study was the lack of a comparison with healthy people. Additionally, the patients we selected were patients with suspected lung cancer who underwent CTC testing and surgical treatment, which resulted in the identification of fewer benign nodules in our patients.

In conclusion, our results suggest that CTC units are feasible diagnostic biomarkers in patients with SPNs. The combination of CTCs with CEA should significantly improve the efficacy of diagnosing malignant nodules. Further investigation into the correlation between preoperative CTC counts and the progression of SPNs is required.

## Data Availability

The datasets obtained and/or analyzed during the current study are available from the corresponding author upon reasonable request.

## Abbreviations

CEA: Carcinoembryonic antigen
CTCs: Circulating tumor cells
GGO: Ground-glass opacity nodules
NSCLC: Non-small cell lung cancer
SPNs: Solitary pulmonary nodules

## References

[1] Hanssen A, Wagner J, Gorges TM (2016). Characterization of different CTC subpopulations in non-small cell lung cancer. Sci Rep.

[2] Tamminga M, Groen HH, Hiltermann TJ (2016). Investigating CTCs in NSCLC-a reaction to the study of Jia-Wei wan: a preliminary study on the relationship between circulating tumor cells count and clinical features in patients with non-small cell lung cancer. J Thorac Dis.

[3] Lorente D, Olmos D, Mateo J (2018). Circulating tumour cell increase as a biomarker of disease progression in metastatic castration-resistant prostate cancer patients with low baseline CTC counts. Ann Oncol.

[4] Aieta M, Facchinetti A, De Faveri S (2016). Monitoring and characterization of circulating tumor cells (CTCs) in a patient with EML4-ALK-positive non-small cell lung Cancer (NSCLC). Clin Lung Cancer.

[5] Messaritakis I, Politaki E, Plataki M (2017). Heterogeneity of circulating tumor cells (CTCs) in patients with recurrent small cell lung cancer (SCLC) treated with pazopanib. Lung Cancer.

[6] Li S, Chen Q, Li H (2017). Mesenchymal circulating tumor cells (CTCs) and OCT4 mRNA expression in CTCs for prognosis prediction in patients with non-small-cell lung cancer. Clin Transl Oncol.

[7] Das M, Riess JW, Frankel P (2012). ERCC1 expression in circulating tumor cells (CTCs) using a novel detection platform correlates with progression-free survival (PFS) in patients with metastatic non-small-cell lung cancer (NSCLC) receiving platinum chemotherapy. Lung Cancer.

[8] Thangavel Hariprasad, Angelis Carmine De, Vasaikar Suhas, Bhat Raksha, Jolly Mohit Kumar, Nagi Chandandeep, Creighton Chad J., Chen Fengju, Dobrolecki Lacey E., George Jason T., Kumar Tanya, Abdulkareem Noor Mazin, Mao Sufeng, Nardone Agostina, Rimawi Mothaffar, Osborne C. Kent, Lewis Michael T., Levine Herbert, Zhang Bing, Schiff Rachel, Giuliano Mario, Trivedi Meghana V. (2019). A CTC-Cluster-Specific Signature Derived from OMICS Analysis of Patient-Derived Xenograft Tumors Predicts Outcomes in Basal-Like Breast Cancer. Journal of Clinical Medicine.

[9] Wang Y, Guo L, Feng L (2018). Single nucleotide variant profiles of viable single circulating tumour cells reveal CTC behaviours in breast cancer. Oncol Rep.

[10] Zong W, Feng W, Jiang Y (2019). Evaluating the diagnostic and prognostic value of serum long non-coding RNA CTC-497E21.4 in gastric cancer. Clin Chem Lab Med.

[11] Kozminsky M, Fouladdel S, Chung JS (2019). Detection of CTC clusters and a dedifferentiated RNA-expression survival signature in prostate Cancer. Adv Sci (Weinh).

[12] Perumal Vanathi, Corica Tammy, Dharmarajan Arun, Sun Zhonghua, Dhaliwal Satvinder, Dass Crispin, Dass Joshua (2019). Circulating Tumour Cells (CTC), Head and Neck Cancer and Radiotherapy; Future Perspectives. Cancers.

[13] Busetto GM, Ferro M, Del Giudice F (2017). The prognostic role of circulating tumor cells (CTC) in high-risk non-muscle-invasive bladder Cancer. Clin Genitourin Cancer.

[14] Sequist LV, Nagrath S, Toner M (2009). The CTC-chip: an exciting new tool to detect circulating tumor cells in lung cancer patients. J Thorac Oncol.

[15] Wei T, Zhu D, Yang Y (2019). The application of nano-enrichment in CTC detection and the clinical significance of CTCs in non-small cell lung cancer (NSCLC) treatment. PLoS One.

[16] Wang Y, Liu Y, Zhang L, et al. Vimentin expression in circulating tumor cells (CTCs) associated with liver metastases predicts poor progression-free survival in patients with advanced lung cancer. J Cancer Res Clin Oncol. 2019. 10.1007/s00432-019-03040-9 PMC6861204 31646374

[17] Keup Corinna, Storbeck Markus, Hauch Siegfried, Hahn Peter, Sprenger-Haussels Markus, Tewes Mitra, Mach Pawel, Hoffmann Oliver, Kimmig Rainer, Kasimir-Bauer Sabine (2019). Cell-Free DNA Variant Sequencing Using CTC-Depleted Blood for Comprehensive Liquid Biopsy Testing in Metastatic Breast Cancer. Cancers.

[18] Wei RR, Sun DN, Yang H (2018). CTC clusters induced by heparanase enhance breast cancer metastasis. Acta Pharmacol Sin.

[19] Tan K, Leong SM, Kee Z (2018). Longitudinal monitoring reveals dynamic changes in circulating tumor cells (CTCs) and CTC-associated miRNAs in response to chemotherapy in metastatic colorectal cancer patients. Cancer Lett.

[20] Gropp C, Lehmann FG, Havemann K (1977). Carcinoembryonic antigen (CEA) in patients with lung cancer: correlation with tumour extent and response to treatment (author's transl). Dtsch Med Wochenschr.

[21] Jia XS (1988). Study of CEA as a marker of cancer and its surrounding tissue in 145 cases of lung carcinoma. Zhonghua Bing Li Xue Za Zhi.

[22] Noge S, Hara N, Ichinose Y (1985). Clinical significance of CEA levels in lung cancer. Gan No Rinsho.

[23] Yang Q, Zhang P, Wu R (2018). Identifying the best marker combination in CEA, CA125, CY211, NSE, and SCC for lung Cancer screening by combining ROC curve and logistic regression analyses: is it feasible?. Dis Markers.

[24] Ost D, Fein AM, Feinsilver SH (2003). Clinical practice. The solitary pulmonary nodule. N Engl J Med.

[25] Aberle DR, Adams AM, Berg CD (2011). Reduced lung-cancer mortality with low-dose computed tomographic screening. N Engl J Med.

[26] Gould MK, Fletcher J, Iannettoni MD (2007). Evaluation of patients with pulmonary nodules: when is it lung cancer?: ACCP evidence-based clinical practice guidelines (2nd edition). Chest.

[27] Liu X, Zhang Z, Zhang B (2018). Circulating tumor cells detection in neuroblastoma patients by EpCAM-independent enrichment and immunostaining-fluorescence in situ hybridization. EBioMedicine.

[28] Ge F, Zhang H, Wang DD (2015). Enhanced detection and comprehensive in situ phenotypic characterization of circulating and disseminated heteroploid epithelial and glioma tumor cells. Oncotarget.

[29] Premasekharan G, Gilbert E, Okimoto RA (2016). An improved CTC isolation scheme for pairing with downstream genomics: demonstrating clinical utility in metastatic prostate, lung and pancreatic cancer. Cancer Lett.

[30] Zhang Z, Ramnath N, Nagrath S (2015). Current status of CTCs as liquid biopsy in lung Cancer and future directions. Front Oncol.

[31] Zhang Z, Shiratsuchi H, Lin J (2014). Expansion of CTCs from early stage lung cancer patients using a microfluidic co-culture model. Oncotarget.

[32] Iwasaki A, Hamanaka W, Hamada T (2007). Comparison between a case-matched analysis of left upper lobe trisegmentectomy and left upper lobectomy for small size lung cancer located in the upper division. Thorac Cardiovasc Surg.

[33] Yu Y, Chen Z, Dong J (2013). Folate receptor-positive circulating tumor cells as a novel diagnostic biomarker in non-small cell lung cancer. Transl Oncol.

